# Diabetic ketoacidosis (DKA) induced cerebral edema complicating small chronic subdural hematoma/hygroma/ at Zewuditu memorial hospital: a case report

**DOI:** 10.1186/s12902-021-00916-1

**Published:** 2022-01-12

**Authors:** Mestet Yibeltal Shiferaw, Tsegazeab Laeke T/Mariam, Abenezer Tirsit Aklilu, Yemisirach Bizuneh Akililu, Bethelhem Yishak Worku

**Affiliations:** Addis Ababa University, school of medicine, Addis Ababa, Ethiopia

**Keywords:** Burr hole, Cerebral edema, Chronic subdural hematoma/Hygroma, Diabetic ketoacidosis, Diabetes mellitus, Tight brain

## Abstract

**Background:**

While both DKA & CSDH/subdural hygroma/ are known to cause significant morbidity and mortality, there is no a study that shows the role & effect of DKA on CSDH/subdural hygroma/ & vice versa to authors’ best knowledge; hence this work will show how important relation does exist between DKA & CSDH/ hygroma.

**Case summary:**

This study highlights the diagnostic & management challenges seen for a case of a 44 years old female black Ethiopian woman admitted with a diagnosis of newly diagnosed type 1 DM with DKA + small CSDH/subdural hygroma/ after she presented with sever global headache and a 3 month history of lost to her work. She needed burrhole & evacuation for complete clinical improvement besides DKA’s medical treatment.

**Conclusion:**

DKA induced cerebral edema on the CSDH/subdural hematoma/ can have a role in altering any of the parameters (except the thickness of CSDH) for surgical indication of patients with a diagnosis of both CSDH +DM with DKA. Hence, the treating physicians should be vigilant of different parameters that suggests tight brain &/ cerebral edema (including midline shift, the status of cisterns, fissures & sulci) and should not be deceived of the thickness of the CSDH/subdural hygroma/alone; especially when there is a disproportionately tight brain for the degree of collection. Whether DKA induced cerebral edema causes a subdural hygroma is unknown and needs further study.

## Background

Both diabetic ketoacidosis and chronic subdural hematoma (CSDH) &/subdural hygroma/ are common medical and surgical problems with significant morbidity & mortality that mandates medical and neurosurgical treatment respectively [1]. Because chronic subdural hematoma is the most common cause of subdural hygroma and might at times be difficult to differentiate one from the other solely on imaging, the term CSDH &/subdural hygroma/ will be used throughout the discussion of this work.

**Fig. 1:**
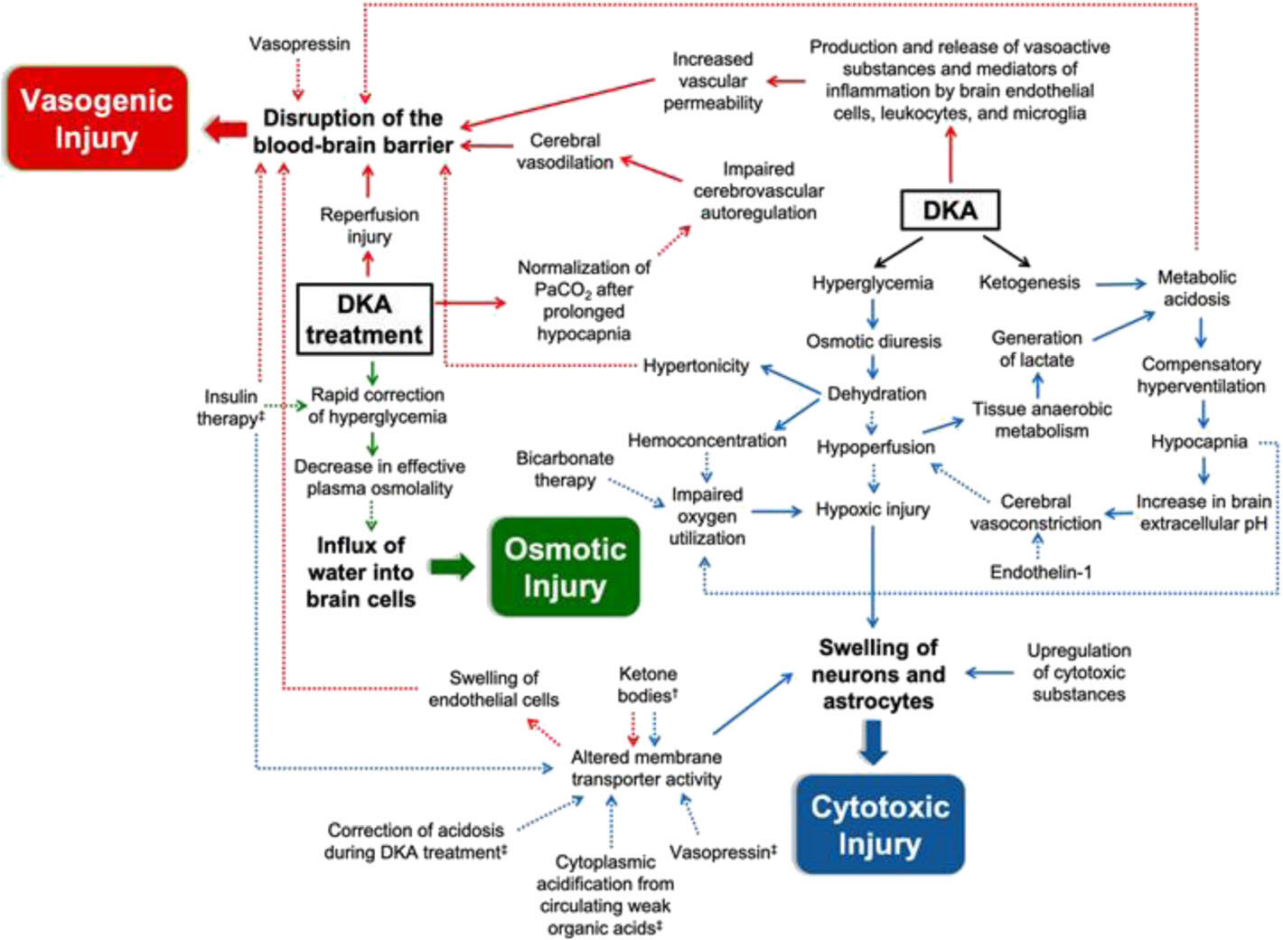
A hypothetical schema of the pathways that contribute to the development of cerebral injury in children with diabetic ketoacidosis. Solid arrows indicate pathophysiologic mechanisms that have been observed in humans. Dashed arrows represent hypothesized mechanisms or those that have only been shown in animal studies. The blue arrows signify factors that lead to cytotoxic injury, including upregulation of cytotoxic substances, altered membrane transporter activity, and hypoxic injury. The red arrows depict mechanisms that predispose towards vasogenic injury, characterized by the disruption of the blood–brain barrier. The green arrows denote treatment-related effects that may cause osmotic injury. A combination of these processes causes cerebral edema in high-risk children. ^†^Factors associated with activation of Na-K-Cl cotransporter in astrocytes and endothelial cells. ^‡^Factors associated with activation of Na+/H+ exchanger in neurons. Abbreviations: DKA, diabetic ketoacidosis ***(Taken from Pediatric Diabetes, Volume: 22, Issue: 2, Pages: 148–160, First published: 16 November 2020, DOI: (***10.1111/pedi.13152***)***

While it is established fact that both diabetic ketoacidosis and CSDH &/subdural hygroma/ can cause tight brain /cerebral edema/ alone, the mechanism how cerebral edema occurs is different. That is, the direct mass effect of the CSDH &/subdural hygroma/ & indirect mass effect from vasogenic edema tend to compress the adjacent sulci, ventricles & basal cisterns and causes cerebral edema if the thickness is significant and it appears to result a proportionate level of brain tightness for the amount of CSDH in majority of cases [2]; while cytotoxic & vasogenic brain edema mechanisms from direct metabolic effects of DKA, water re-distribution effect of DKA & treatment related changes seen in DKA (see Fig. 1) are responsible for cerebral edema from DKA [3–6].

To best of authors’ knowledge, this is the first article which presents a case of cerebral edema in a patient who presented with a diagnosis of both DKA + CSDH. This paper tries to show whether or not the severity of brain tightness (edema) seen in our patient is from the direct mass effect of CSDH &/subdural hygroma/ or from DKA related cerebral alteration due to the presence of DKA. It also highlights if there is any difference in the management of patients having CSDH &/subdural hygroma/ alone from those having CSDH &/subdural hygroma/ with DKA.

## Case presentation

### History

This is a 45 years old female black Ethiopian newly diagnosed type 1 DM patient who first presented with diabetic ketoacidosis which was diagnosed in a routine investigations done for a compliant of sever global headache of 1 month duration following a 1 month prior history of trivial trauma she sustained to her head. The headache was sever and persistent for the whole duration of one month but get worsened & unbearable for 1 week before current visit. In association, she had history of intermittent vomiting of ingested matter of week duration. For the above complaints, she visited multiple health facility and received pain killer medications though there was no improvement. Otherwise, she has no weakness, abnormal body movement and loss of consciousness. She also had no the classic presentation of DM and DKA as well. No known precipitants identified for her DKA except the trivial trauma history she had. Apart from the trivial trauma history, she did not have risk factors for CSDH, including history of use of medications that increase bleeding risk. No previous surgery & other comorbidities. No family history of chronic medical illnesses including diabetes mellitus.

### Physical finding

She looked acutely sick looking from due to sever global headache she was suffering from. She had tachypnea and tachycardia which was presumed to be due to the severe headache. She was afebrile and had normal blood pressure limit. Buccal mucosa was dry. Air entry to the lung fields was good bilaterally. Neurologic exam was also unremarkable.

### Laboratory

Her basic lab investigations including CBC, RFT, LFT, serum electrolytes and her coagulation profiles were with normal range. But, her fasting blood sugar was 530 mg/dl with + 2 ketonuria and + 3 glucosuria, hence the diagnosis of type 1 DM with DKA was diagnosed.

### Imaging

Non contrast brain CT scan was obtained with possible impression of extra axial collection as she had trivial trauma history as a risk factor for the cause of headache before the commencement of treatment for DKA.

**Fig. 2:**
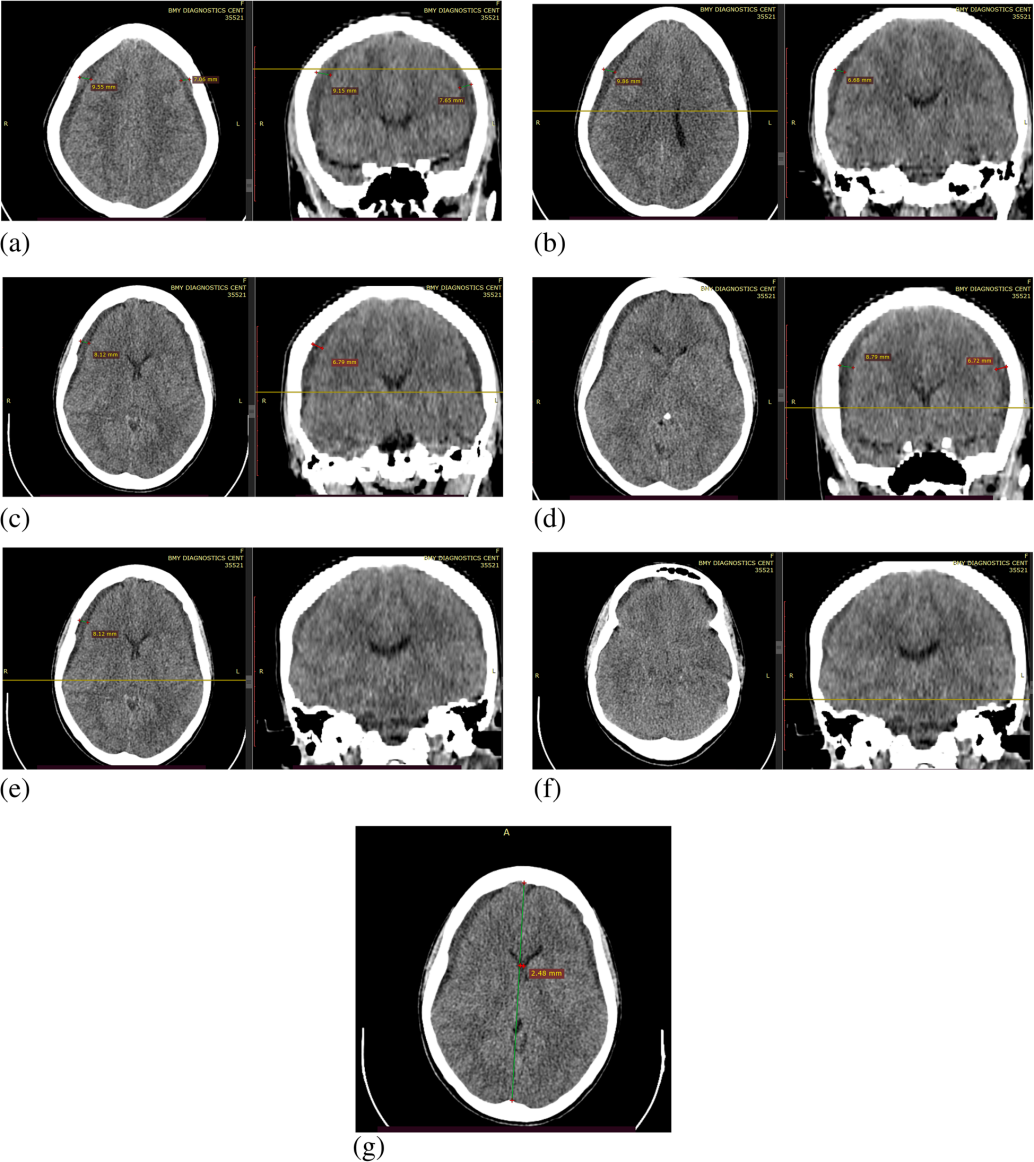
(image of patient CT scan of the patient at different levels of section as labeled from ‘a to g’)

Accordingly, the brain CT showed a bilateral fronto-parietal subdural hypodense extra-axial collection with a maximum thickness of 9.86 mm & 7.65 mm on the right and left side respectively. In addition, there is global effacement of hemispheric sulci, sylvian fissure, basal cisterns & ventricles. The gray white matter differentiation is also poor and the intracranial fosses appear crowded. There is no significant midline shift however (< 3 mm). A likely diagnosis of chronic subdural hematoma with DDX of subdural hygroma was made based on the preoperative imaging (See Fig. 2 for the CT scan of the patient below at different levels of sections in the image from ‘a to g’).

### Diagnosis

Based on the lab and imaging evidences, the diagnosis of CSDH + R/O subdural hygroma + newly diagnosed Type 1 DM with DKA + cerebral edema 20? (Mass effect from CSDH +? /DKA induced) was made.

### Treatment

Since our patient had both medical and neurosurgical problem, multidisciplinary team was involved it her management. Accordingly, both medical & neurosurgical treatment was given by the respective team of physicians.

#### Medical management

Accordingly, the fluid deficit, potassium deficit and the hyperglycemia was corrected using normal saline, potassium replacement and regular insulin administration as per diabetic keto-acidosis management protocol. Patient then followed with an hourly basis of RBS and ketone urine with clinical response of the patient. The patient became ketone free after 6 h of management with the level of hyperglycemia reduced to 250-300 mg/dl. Despite our patient had metabolic response, her headache persisted and didn’t improve with the medical management.

#### Surgical management

Once the patient was stabilized metabolically &/medically, neurosurgical intervention was decided to be made as the patient has tight brain which is disproportionate for the thickness of the extra-axial collection as detailed in the CT finding.

Written informed consent was obtained for her to undergo surgery and for her case summary can be published for educational purpose. Patient was then taken to the operating room and positioned supine. Patient put on intranasal oxygen, and sedation with both pethidine and diazepam achieved. Then hair was shaved and local anesthesia using 2% lidocaine administered at the surgical site. Surgical field cleaned with alcohol first which then painted with providone iodine. Proper prepping and draping then done. A 3 cm sized skin incision at the epicenter (maximal thickness) of the extra-axial collection done; which is at the coronal suture and 5 cm off of midline and burr hole was made using manual bur. Bone dust thoroughly irrigated. Dural vessels cauterized with bipolar cautery forceps and dura opened in a cruciate fashion on the right side first and left side then after. Dural lips opened in a cruciate fashion tucked up with a bipolar cautery. Upon opening the dura a xanthocromic CSF appearing fluid come out with moderate pressure bilaterally (more on right). There was no characteristic dark motor oil appearing hemolyzed blood expected to be seen in CSDH. Copious irrigation with saline done; after which subdural drain left bilaterally and skin closed in a locking fashion bilaterally. Meticulous hemostasis secured with bipolar cautery in each step of the procedure. Patient transferred with stable vital signs to post anesthesia recovery unit and final postoperative diagnosis of Type 1 DM with DKA + Subdural hygroma likely from CSDH + DKA induced cerebral edema was made.

### Follow-up & treatment response

Patient postoperatively was put on analgesics and sliding scale of insulin management. Her headache responded well after the surgical management was done. She was also followed with random blood sugar & urine ketone evaluation every 6 h and never returned back to DKA in the postoperative period. Drain removed after 24 h. Patient then was discharged improved on her 3rd postoperative day after the patient was linked to medical side for comprehensive diabetic care.

## Discussion

Generally, cerebral edema though more common in children, it is also reported to occur in young adults [3] like ours and carries a mortality rate of 21–25% [7]. The risk of cerebral edema is related to the severity and duration of DKA. It is often associated with ongoing hyponatremia. Cerebral edema is correlated with the administration of bicarbonate. Concerns about the role of overaggressive or overly hypotonic fluid resuscitation as a cause of the edema that have been raised in the past correlate more closely with disease severity than with rapid administration of fluids. Accordingly, patients with fluid refractory shock and azotemia at admission had higher odds for development of cerebral edema [8]. That our patient has a protracted symptomatology might contribute for the DKA induced cerebral edema.

Unique to our patient, in contrast to other case reports with cerebral edema in DKA patients, is the presence of CSDH/subdural hygroma/. Even though the tight brain seen in our patient can partly be attributable to the direct mass effect of CSDH/subdural hygroma/, the degree of brain tightness and cerebral edema is so disproportionate for the degree of the extra-axial collection she had; and hence, DKA induced cerebral edema must have a role in partly contributing the brain disproportionately tight. Similarly, while the trivial trauma could have caused the CSDH/subdural hygroma/ in our patient, it is unknown whether or not the DKA induced cerebral edema caused it.

So, it can be concluded that, as diabetes by itself independently increases the incidence of CSDH compared to non-diabetics by a factor of 1.57 [9], diabetes and its complication DKA portends a higher risk of mortality because of the added mass effect of DKA induced cerebral edema on the CSDH/subdural hematoma/. Hence, whether surgical intervention is needed for CSDH/subdural hygroma/ or not is a function of the thickness, midline shift, the status of cisterns, fissures & sulci, it is worth full to underscore the possible existence of DKA induced cerebral edema & its role in altering any of the parameters (except the thickness) for surgical indication of patients with a diagnosis of both CSDH +DM with DKA. A lower threshold for surgical intervention might be needed in that case though more study is needed. While this work is written after prospectively by the managing clinicians, its clinical summary is reliable and described as strength; its limitation being the case is only one.

## Conclusion

DKA induced cerebral edema on the CSDH/subdural hematoma/ can have a role in altering any of the parameters (except the thickness) for surgical indication of patients with a diagnosis of both CSDH +DM with DKA. Hence, the treating physicians should be vigilant of this and should not be deceived of the thickness of the CSDH/subdural hygroma/alone especially when there is a disproportionately tight brain for the degree of collection. Whether DKA induced cerebral edema causes a subdural hygroma is unknown and needs study.

## Data Availability

Full supporting data is available in the hand of correspondent author, and will be available when needed.

## Abbreviations

CBC: Complete Cell Count
CSDH: Chronic Subdural Hematoma
CSF: Cerebrospinal Fluid
CT: Commuted Tommography
DDX: Differential Diagnosis
DKA: Diabetic Ketoacidosis
DM: Diabetes Melitus
LFT: Liver Function Test
RFT: Renal Function Test
